# Supplementary material for: QseBC is involved in the biofilm formation and antibiotic resistance in Escherichia coli isolated from bovine mastitis
Source: PeerJ. 2020 Mar 25;8:e8833. doi: 10.7717/peerj.8833 (PMC7102498; doi:10.7717/peerj.8833)
Supplement: Supplemental Information 1 [file peerj-08-8833-s001.zip › Supplemental Dataset Files/Supplemental Figure/Figure S1 legend.docx]

**Figure S1. PCR identification of strains XL4/pCqseBC, XL4/pSTV28 and WT/pSTV28 using M13 primers.** M: marker; Line 1: The band of XL4/pCqseBC; Line 2: The band of XL4/pSTV28; Line 3: The band of WT/pSTV28.
