# Supplementary figures and images for: QseBC is involved in the biofilm formation and antibiotic resistance in Escherichia coli isolated from bovine mastitis
Source: PeerJ. 2020 Mar 25;8:e8833. doi: 10.7717/peerj.8833 (PMC7102498; doi:10.7717/peerj.8833)

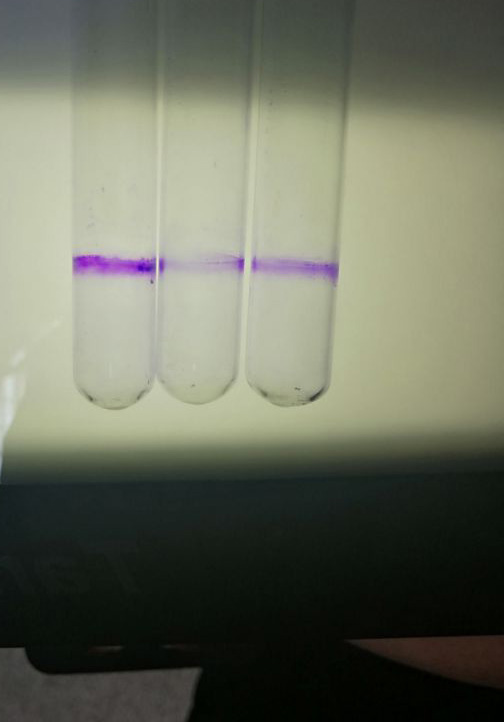

Supplement: Supplemental Information 1 [file peerj-08-8833-s001.zip › Supplemental Dataset Files/Raw Data/File3/biofilm-1.jpg]

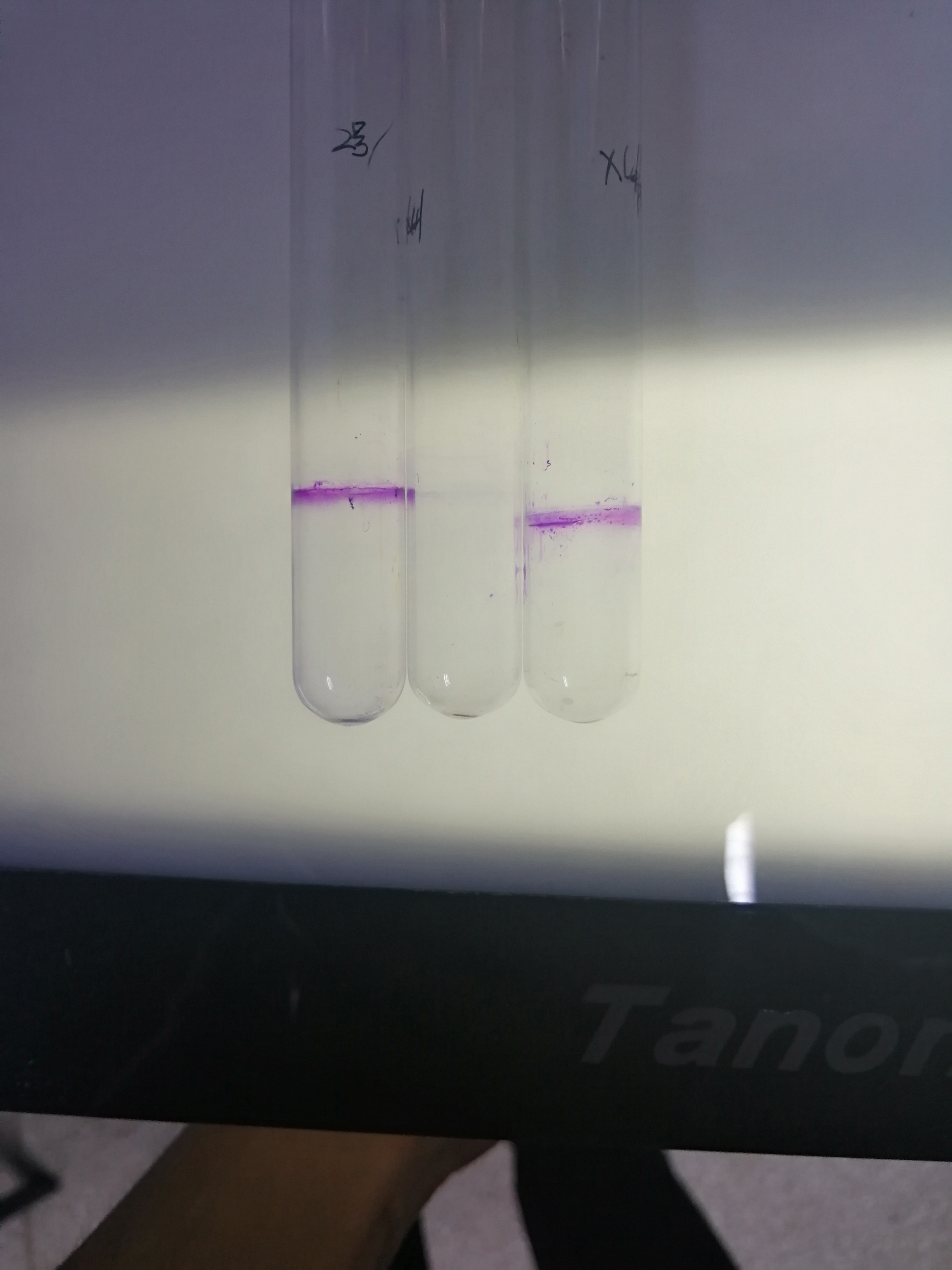

Supplement: Supplemental Information 1 [file peerj-08-8833-s001.zip › Supplemental Dataset Files/Raw Data/File3/biofilm-2.jpg]

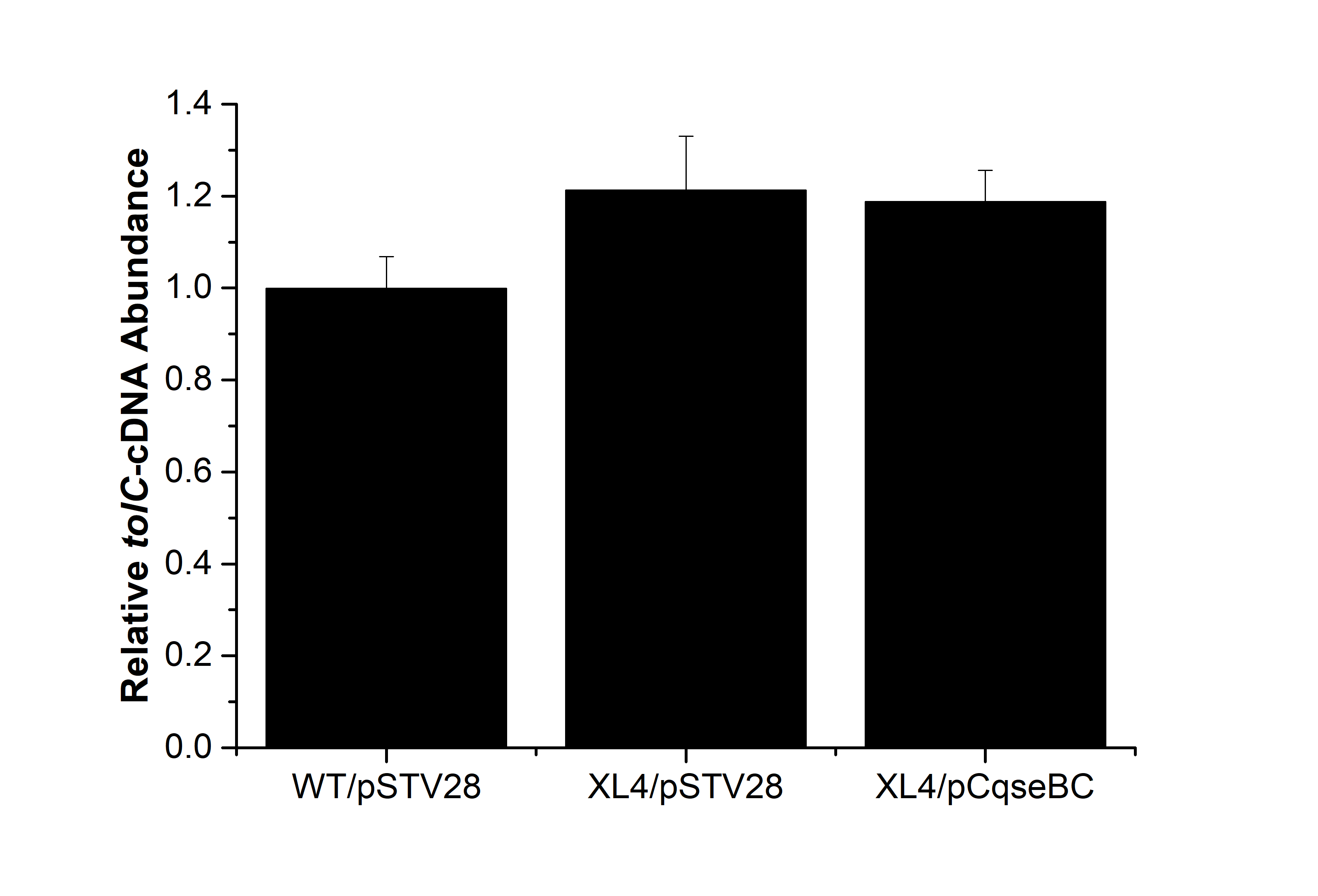

Supplement: Supplemental Information 1 [file peerj-08-8833-s001.zip › Supplemental Dataset Files/Raw Data/File6/tolC.png]

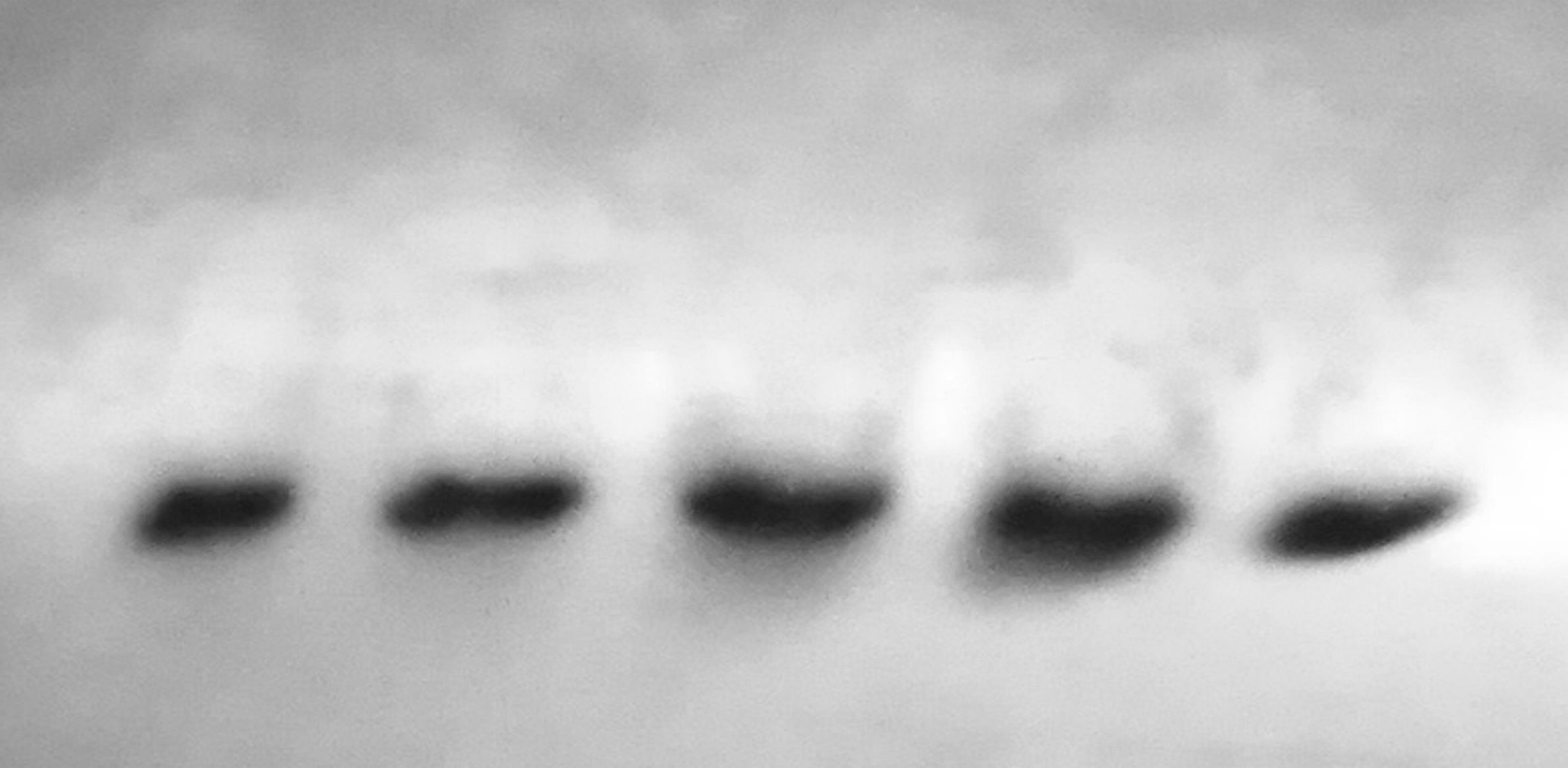

Supplement: Supplemental Information 1 [file peerj-08-8833-s001.zip › Supplemental Dataset Files/Raw Data/File7/acrA.jpg]

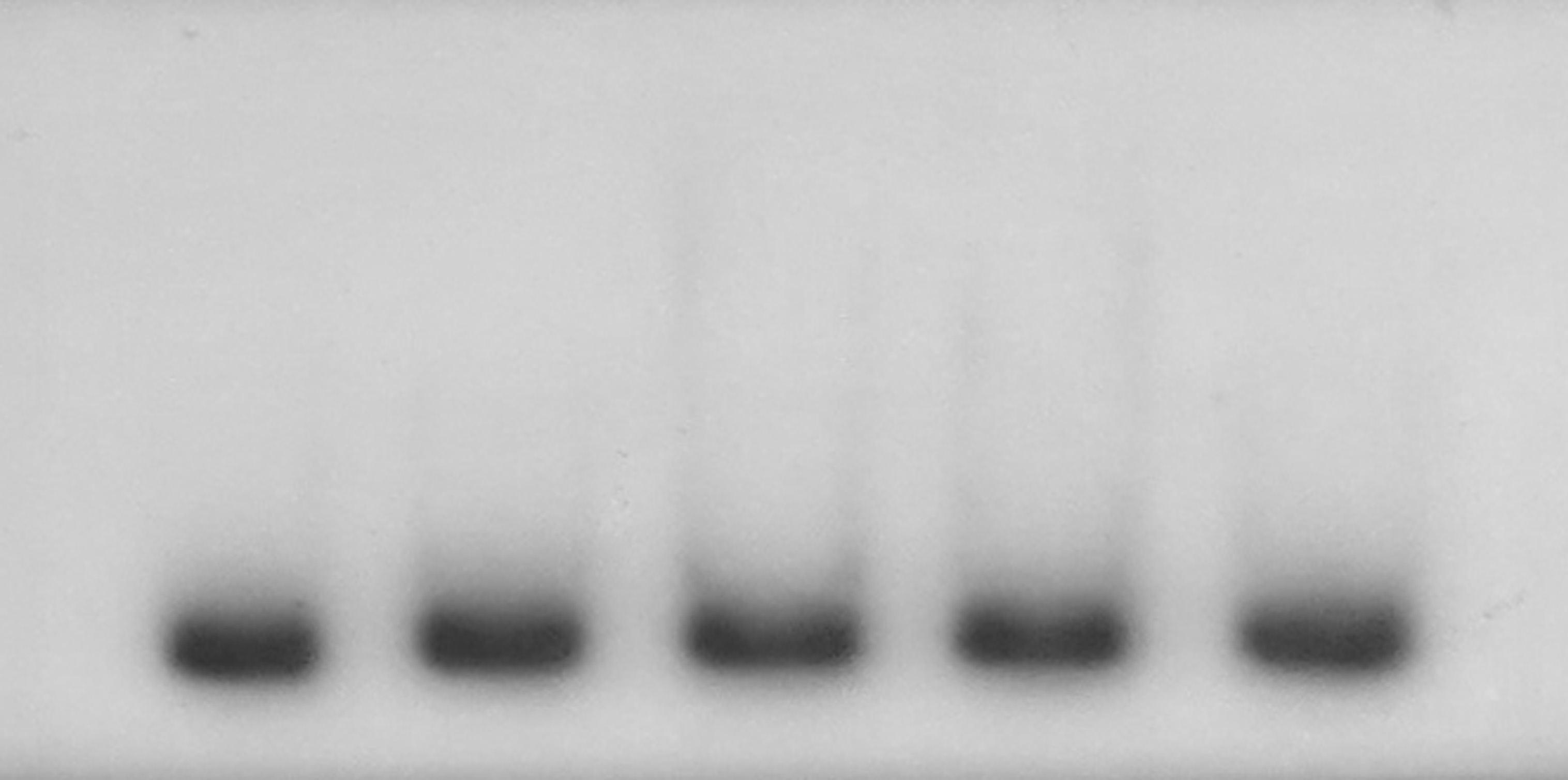

Supplement: Supplemental Information 1 [file peerj-08-8833-s001.zip › Supplemental Dataset Files/Raw Data/File7/acrD.jpg]

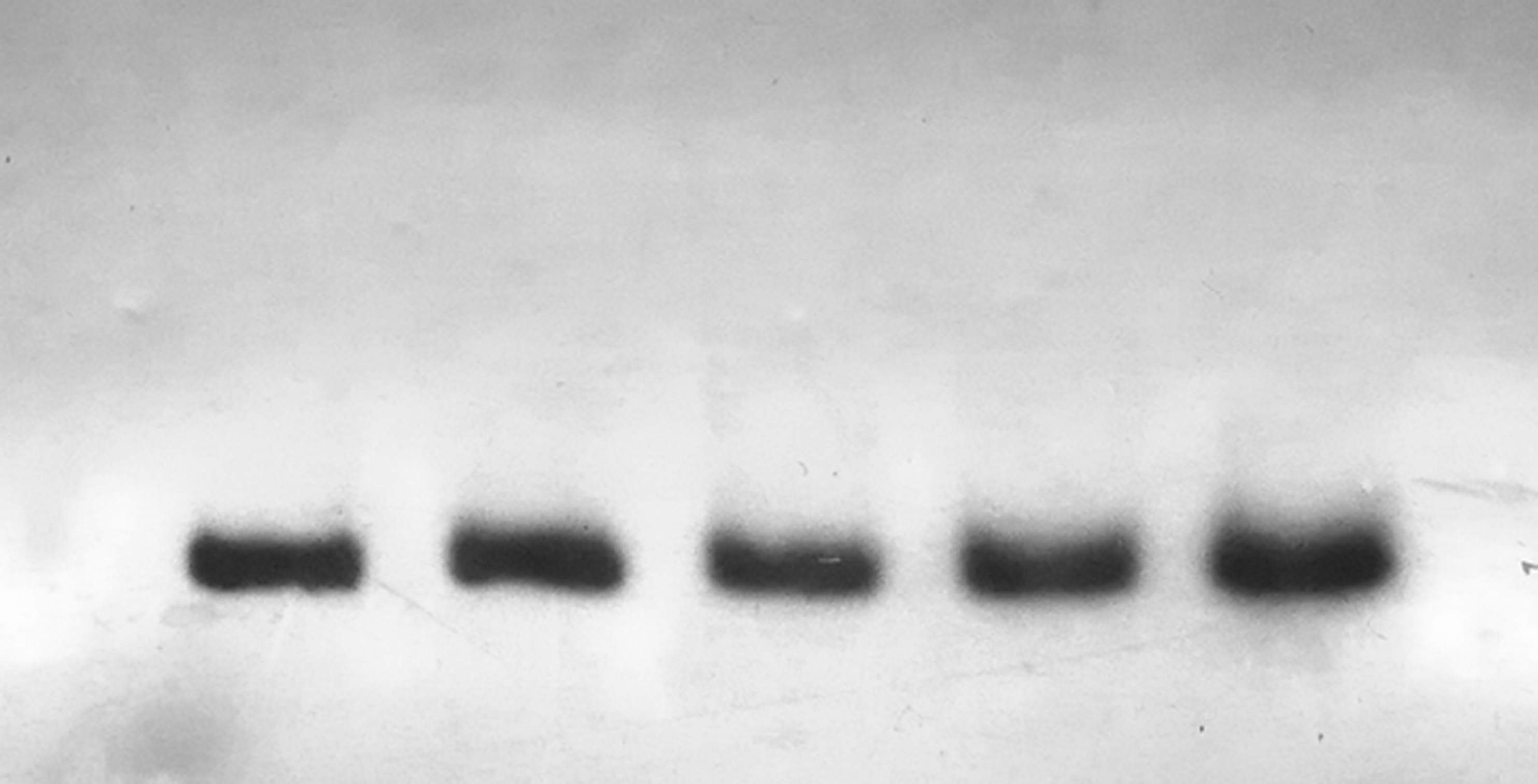

Supplement: Supplemental Information 1 [file peerj-08-8833-s001.zip › Supplemental Dataset Files/Raw Data/File7/emrD.jpg]

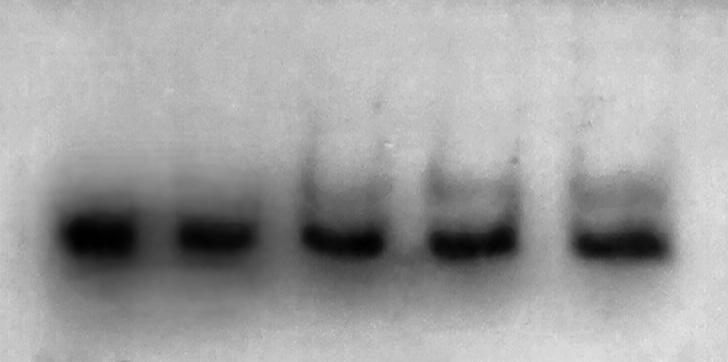

Supplement: Supplemental Information 1 [file peerj-08-8833-s001.zip › Supplemental Dataset Files/Raw Data/File7/marA.jpg]

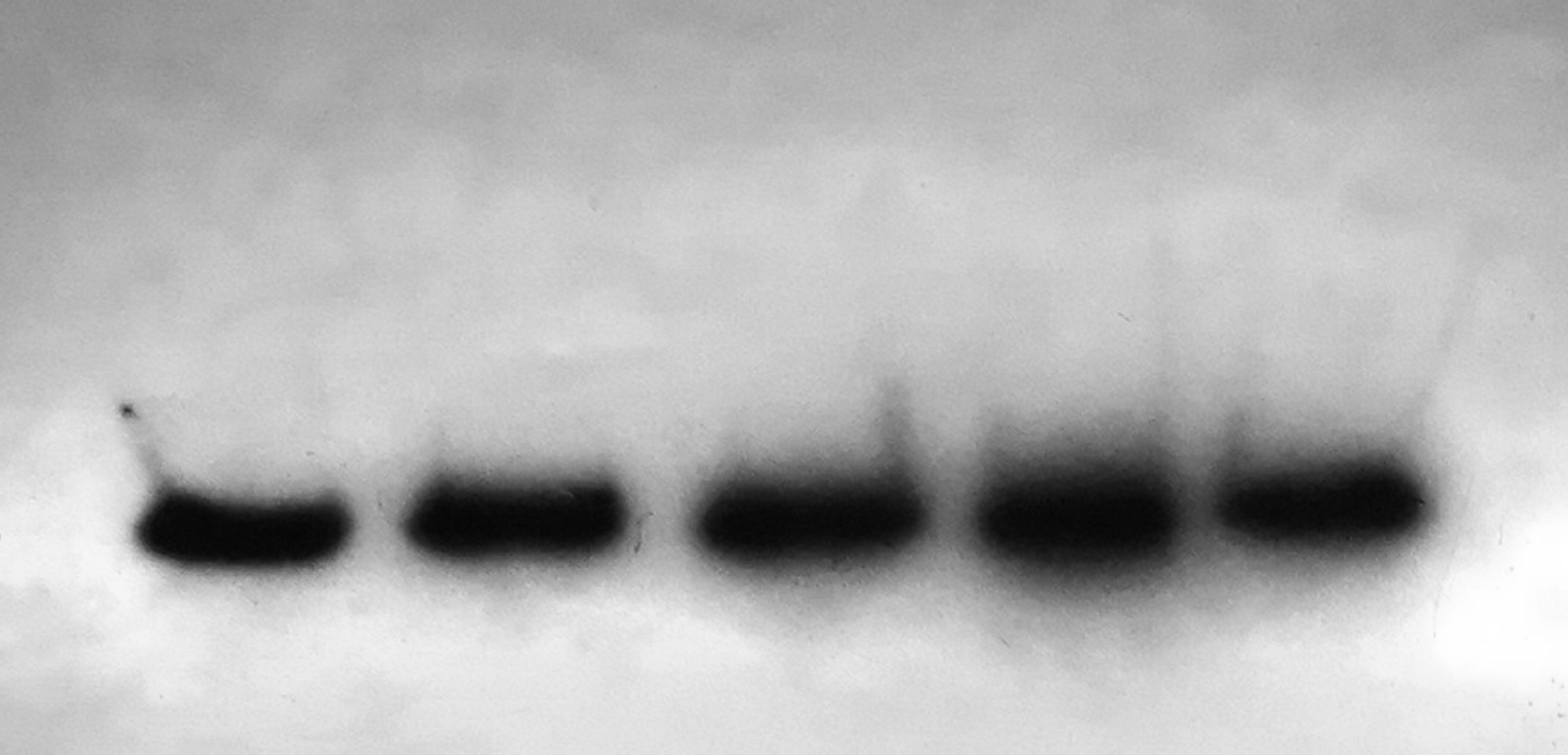

Supplement: Supplemental Information 1 [file peerj-08-8833-s001.zip › Supplemental Dataset Files/Raw Data/File7/mdtH.jpg]

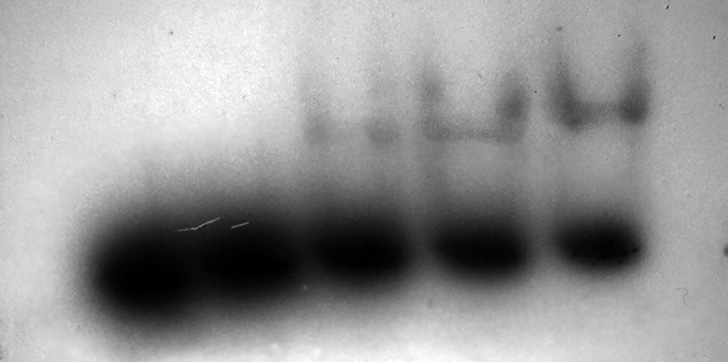

Supplement: Supplemental Information 1 [file peerj-08-8833-s001.zip › Supplemental Dataset Files/Raw Data/File7/qseB.jpg]

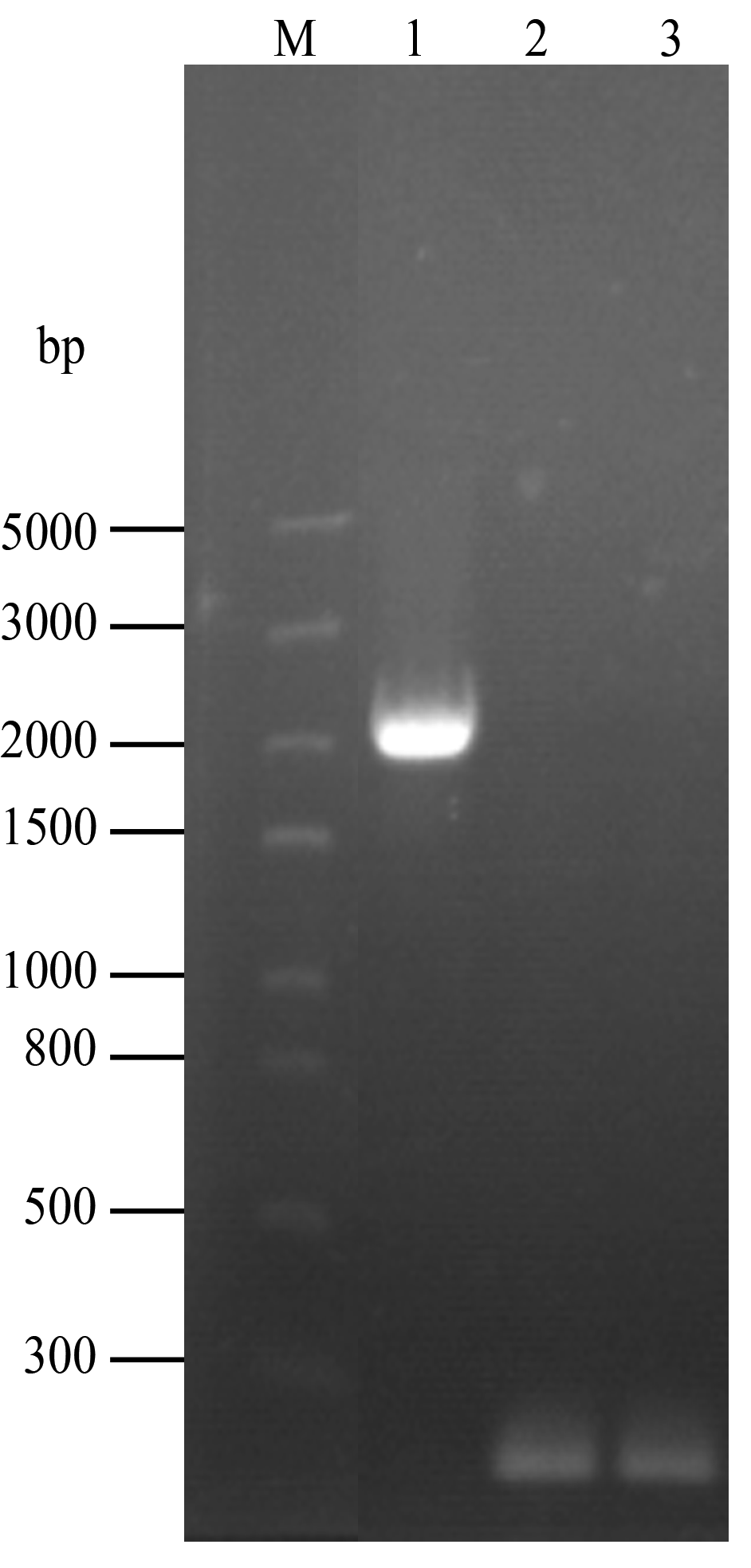

Supplement: Supplemental Information 1 [file peerj-08-8833-s001.zip › Supplemental Dataset Files/Supplemental Figure/Fig S1.png]
